# Perspectives and attitudes of breastfeeding women using herbal galactagogues during breastfeeding: a qualitative study

**DOI:** 10.1186/1472-6882-14-216

**Published:** 2014-07-02

**Authors:** Tin Fei Sim, H Laetitia Hattingh, Jillian Sherriff, Lisa B G Tee

**Affiliations:** 1 School of Pharmacy, Faculty of Health Sciences, Curtin University, Perth, Western Australia, Australia; 2 School of Public Health, Faculty of Health Sciences, Curtin University, Perth, Western Australia, Australia

**Keywords:** Herbal galactagogues, Breastfeeding women, Lactation, Perspectives, Fenugreek

## Abstract

**Background:**

Some herbal galactagogues have gained reputation and recognition by the public and health professionals as alternative approaches to increase breast milk supply. This study explores the perspectives and attitudes of breastfeeding women towards the use of herbal galactagogues while breastfeeding, their experiences, and why and how they have chosen an alternative option over conventional treatments to enhance breastfeeding performance.

**Methods:**

This exploratory research was conducted through in-depth semi-structured interviews with women living in Perth, Western Australia, who were using one or more herbal galactagogues during breastfeeding. Purposeful and subsequent snowball sampling methods were employed to recruit participants. All interviews, facilitated by an interview guide, were audio-recorded, then transcribed verbatim. Thematic analysis was used to analyse qualitative data to construct themes and subthemes.

**Results:**

The perspectives and attitudes of the 20 participants are classified under three main headings: i) use of herbal medicines during breastfeeding, ii) available herbal medicines resources, and iii) level of breastfeeding support received. Throughout the interviews, participants described how their perseverance and determination to breastfeed, as well as concerns over breastfed infants’ safety with conventional treatments, influenced their choice of therapy. A sense of self-efficacy and autonomy over their own health needs was seen as influential to their confidence level, supported self-empowerment and provided reassurance throughout the breastfeeding journey. There was also a desire for more evidence-based information and expectations of health professionals to provide credible and reliable information regarding the use of herbal medicines during breastfeeding.

**Conclusions:**

This study has enhanced our understanding of the perspectives and attitudes of breastfeeding women towards the use of herbal medicines, in particular galactagogues, while breastfeeding. The positive attitudes of breastfeeding women identified in this study highlight the need for further research into evaluating the safety and efficacy of commonly used herbal galactagogues, whilst the negative views on breastfeeding education should be taken into consideration when implementing or improving breastfeeding-related health policies.

## Background

The use of complementary and alternative medicines (CAM), including herbal medicines, amongst the general population is increasing worldwide [1-3]. Garlic, green tea, aloe vera, chamomile and echinacea are commonly used herbs with potential medicinal properties [3,4]. Previous studies have highlighted the popularity of these herbs and herbal medicines amongst the general population as well as breastfeeding women [5-9]. Some herbal medicines including galactagogues have indeed gained some reputation and recognition by the public and some health care providers, for example naturopaths, as alternative approaches to enhance breastfeeding performance [9-11].

A recent prevalence study in Western Australia which involved surveying women who were breastfeeding in the previous 12 months, revealed that 59.9% of the 304 survey respondents reported the use of at least one herbal medicine whilst breastfeeding, with the top ten most commonly used being fenugreek (*Trigonellafoenum*-*graecum*), ginger (*Zingiber officinale*), dong quai (*Angelica sinensis*), chamomile (*Matricaria chamomilla*), garlic (*Allium sativum*), blessed thistle (*Cnicus benedictus*), cranberry (*Vaccinium macrocarpon*), fennel (*Foeniculum vulgare*), aloe vera and peppermint [5]. The same study reported that 24% of respondents were using herbal medicines for the purpose of increasing breast milk supply and to promote breastfeeding performance, regardless of whether participants had been diagnosed with insufficient milk supply or not [5]. Fenugreek was the most commonly used herbal galactagogue during breastfeeding amongst the survey respondents. Other commonly reported herbal galactagogues included blessed thistle (*Cnicusbenedictus*), fennel (F*oeniculumvulgare*), goat’s rue (*Galegaofficinalis*), nettle (*Urticadioica*), blackthorn berry (*Prunus spinosa*), and shatavari (*Asparagus racemosus*) [5].

Despite the many efforts to facilitate breastfeeding, some women may still experience difficulty with breastfeeding due to numerous factors. For example: many societal and environmental factors such as cultural norms, hospital, home, work and community environments have been shown to impact on the rate of successful breastfeeding [12]. Another commonly reported reason for unsuccessful breastfeeding or early weaning is perceived low or insufficient breast milk supply [13]. Poor breastfeeding technique or latching leading to ineffectual milk removal, deficient mammary gland tissue and maternal hormonal imbalances can all contribute to insufficient supply of milk [14,15]. Once these issues have been addressed and other strategies have been followed, such as education about techniques by lactation consultants, and milk flow remains insufficient, galactagogues could be trialled [16].

Although certain herbs have a long history of being used as galactagogues, scientific evaluation is lacking to verify the clinical efficacy of most of these herbs [9]. However, many women continue to use herbal galactagogues based on anecdotal evidence [9,10]. Research into the potential impact on successful breastfeeding and perceived efficacy or psychological benefits of herbal galactagogues would provide insights into the use of these galactagogues as alternative options for breastfeeding women who wish to increase their breast milk supply. Gaining an understanding of breastfeeding women’s perspectives, why and how they have chosen to use herbal galactagogues over conventional options to increase breast milk supply, their experiences and the factors or indicators that influenced their breastfeeding performance, will provide insight into the potential value of herbal galactagogues and identify research gaps to guide direction of future studies.

This study aimed to obtain an understanding of the perspectives and attitudes of women towards the use of herbal galactagogues whilst breastfeeding. The study also aimed to explore why women have chosen alternative remedies to promote breastfeeding and what factors were influencing their decision-making. A better understanding of their unique needs and perspectives will enrich our knowledge in the area of CAM and in turn benefit women who need to use medicines during breastfeeding.

## Methods

This study was approved by the Human Research Ethics Committee of Curtin University (approval number HR85/2012).

### Study design

This study involved exploratory research conducted through in-depth semi-structured interviews with women who were using one or more herbal galactagogues during breastfeeding to increase breast milk supply and facilitate breastfeeding. An interview guide with a mix of ten closed and seven open-ended questions was used during the interviews to obtain information regarding the pattern of use of herbal galactagogues, and explore the perspectives and attitudes towards the use of these medicines during breastfeeding, as below:

– Questions one to eight focused on documenting the pattern of use and types of herbal galactagogues, dosage forms and administration, reasons for use and source of recommendation,

– Questions nine to 11 focused on exploring the perceived efficacy and safety based on participants’ personal experience,

– Questions 12 to 17 (including sub-questions) focused on participants’ general perspectives and attitudes towards the use of herbal medicines during breastfeeding, role of health professionals and the availability of information or resources.

### Participants and recruitment

Breastfeeding women living in the Perth Metropolitan area were invited to participate. All participants had to: i) be 18 years or older, ii) be breastfeeding or have breastfed in the previous 12 months, and iii) previously have used or were using one or more herbal medicines as galactagogues during breastfeeding to increase breast milk supply or to improve breastfeeding performance. Participants were not required to have been diagnosed with insufficient milk supply and could have been from any cultural or ethnic background.

Purposeful sampling and subsequent chain or snowball sampling methods were used to recruit participants, specifically targeting breastfeeding women who visited naturopaths or who had used herbal medicines. Purposeful method of sampling was considered most appropriate as it allows careful selection of cases which are information-rich to facilitate comprehensive qualitative data to be collected, which in turn improves credibility and reliability of the research findings [17].

Participants were initially recruited from naturopathic clinics with a focus on CAMs use and breastfeeding where posters with details of the study were displayed. The study was also advertised to the wider public through promotion in local health and parenting papers and the Curtin FM 100.1 Perth radio station. Interested women were encouraged to contact the researcher to express their interest. Subsequently, a snowball sampling method was adopted for further recruitment where participants were requested to share the study information with other breastfeeding women. Through the snowballing effect, two community pharmacies with a focus on breastfeeding and naturopathy were identified and approached for participant recruitment purposes. Study posters were subsequently displayed at the pharmacies.

Portney and Watkins [18] commented that sample size determination in qualitative research is based on experience, judgment and the research purpose. According to these authors [18], “samples that are too small will not support claims of having reached a point of data saturation. Samples that are too large will not permit the in-depth analysis that is the essence of qualitative inquiry”. Based on a similar galactagogue study involving 23 participants in British Columbia [14], a decision was made to initially recruit up to 20 women and through the data analysis determine whether a point of saturation was reached, when no new themes emerged. Guest *et al*. [19] studied the variability and degree of data saturation in qualitative research and reported that data saturation took place within the first twelve in-depth interviews. Twenty participants were therefore considered sufficient to reach data saturation, as no new themes had merged, whilst also enabling in-depth analysis.

### Data collection

Prior to conducting the interviews, an information sheet about the research and what the interview would cover was provided to participants. For the face-to-face interviews, participants were requested to sign a consent form prior to conducting the interviews. An electronic copy of the participant information sheet was emailed to participants who had provided their email contact and requested a telephone interview. In cases where email contact was not available, the content of the participant information sheet was read to the participant over the telephone and verbal consent was obtained before the interview. Participants were given ample opportunity to ask questions and were reminded that the study was completely voluntary and that they could withdraw at any stage without prejudice.

Taking into consideration the variability between participants and at the same time ensuring that the topic of discussion could be thoroughly covered, the interviewer (TFS) followed a flexible and discrete approach throughout the process. Transgression from the interview guide was necessary in some instances for example when the interviewee mentioned an issue not necessarily addressing the questions in the guide, but still considered relevant to the topic. All interviews were audio-recorded and subsequently manually transcribed verbatim.

### Data analysis

The transcripts were analysed using descriptive and qualitative approaches. Participants were de-identified and codes were used in the analysis (the first interviewee was BW1 for Breastfeeding Woman 1). This paper reports on the qualitative findings of this study, using thematic analysis as described by Boyatzis [20]. In order to achieve thorough understanding of the themes, contents of the transcripts were read repeatedly by the primary investigator (TFS). The emerged ideas or topics along with their supporting quotes were documented. These topics were then grouped and reclassified as subthemes. The process continued until all subthemes were regrouped to form major overall themes. Throughout the process of analysis, project supervisors (LT, LH) provided input and reviewed the themes to ensure reliability.

## Results

A total of 20 breastfeeding women living in the Perth metropolitan area who were using herbal galactagogues were interviewed between October 2012 and April 2013. All interviews were conducted on a one-to-one basis: ten face-to-face at a place convenient to the participant and ten via telephone. Of the 20 participants, one was of Middle-Eastern descent, two were of Asian descent, and the other 17 were of Caucasian descent. Interviews ranged between 18 to 78 minutes with a median duration of 34 minutes. All participants had used fenugreek during breastfeeding, with three of them having used a combination of fenugreek and blessed thistle, and seven having used naturopaths’ own ‘lactation tincture’ containing a combination of herbal ingredients.

Table 1 provides a summary of the themes and subthemes that emerged during data analysis with the major themes being: 1) reasons for the use of herbal medicines during breastfeeding, 2) the need for herbal medicines resources and 3) the level of breastfeeding support identified.

**Table 1:** **Perspectives and attitudes of women**: **major themes and sub**-**themes**

**Major themes**	**Sub-themes**
1. Reasons for the use of herbal medicines	Perseverance and determination to breastfeed
	Confidence, self-empowerment and reassurance
	Concerns over breastfed infants’ safety
	Role and expectation of health care workers
	Parental and peer influence
2. The need for herbal medicines resources	Information needs
	Credibility and reliability of information
	Expectations of health professionals
3. The level of breastfeeding support	Positive feedback
	Negative feedback

### Use of herbal medicines during breastfeeding

Participants reported four main reasons for the use of herbal galactagogues, namely perceived insufficient milk supply, diagnosed insufficient milk supply, as a supplement and as part of the tradition. Besides those who had been diagnosed with insufficient milk supply by health professionals, all other participants embraced the “just-in-case” approach to use herbal galactagogues prophylactically in order to avoid breast milk supply issues. Five subthemes about the use of herbal medicines during breastfeeding were identified as discussed below with selected quotes to assist with explanation of the concepts.

#### ***Perseverance and determination to breastfeed***

All participants seemed to have adopted the ‘breast is best’ philosophy. These women acknowledged and appreciated the health, physical and psychological benefits of breastfeeding to both mothers and infants.

“…*how much it helped my bonding with my baby and even just a general satisfaction from it*, *not only satisfaction but also I would say the benefits were much higher*”. (*BW 1*).

Recognition of the importance and significance of breastfeeding was identified as the main facilitator to develop perseverance and a determined attitude to breastfeed:

“… *basically I was just trying to get him back to the breasts and don*’*t want to stop breastfeeding him*, *so it*’*s to try and increase the supply because there is a need there*”. (*BW 11*).

All participants were familiar with the recommendation of the Australian Dietary Guidelines 2013 to breastfeed exclusively for the first six months of an infant’s life [21]. Taking into consideration the advantages of breastfeeding along with endorsement from their health professionals, perseverance and determination played a vital role in successful breastfeeding as the women were prepared to take all required actions to avoid the use of infant formula as much as possible.

“*Breastfeeding is not easy*, *definitely not. You need to persevere and you need to be absolutely patient with everything that you do. You need to have very high patience level and you need to feel comfortable and confident doing it*”. (*BW 20*).

An underlying theme was observed when women described their strong mental will to breastfeed.

“*I mean honestly*, *if drinking snake oil would make me have more breast milk I would have done it*, *anything that helps*!” (*BW 3*).

“*I certainly am not opposed to the idea of using herbs to support breastfeeding. Really*, *I don*’*t care what it is*, *as long as I can tolerate it and it helps*, *I am willing to try anything*”. (*BW 18*).

#### ***Confidence, self-empowerment and reassurance***

There seemed to be a relationship between the women’s breastfeeding confidence level and duration of exclusive breastfeeding. Even in the absence of milk volume measurement, one participant described how the use of herbal galactagogues promoted her confidence and fostered self-empowerment to breastfeed.

“…*because this* [*fenugreek*] *works so quickly and it just gave me that confidence straight away. It took away that anxiety and stress*”. (*BW 6*).

Many participants also mentioned the feeling of reassurance through the use of herbal supplements during breastfeeding, which was especially important for first-time mothers. Hence, the use of herbal galactagogue was described as a method of reassurance in the context of their own perceptions. The positive emotional impact contributed to the success of breastfeeding practices amongst the participants.

“*I think it*’*s* [*fenugreek*] *worth trying. And as for me*, *I certainly find that useful and reassuring that I have found something effective to increase my milk supply. As a new mum*, *you just never know*, *you never know what is coming*, *what problems you will encounter and I certainly did not anticipate that milk supply will be an issue. I have always thought that breastfeeding is easy and will come naturally because everyone else does it*, *and I wasn*’*t told about it being an issue*”. (*BW 12*).

#### ***Concerns over breastfed infants’ safety***

A number of participants voiced their concerns over breastfed infants’ safety and the use of medicine by the mother. These participants were cautious and apprehensive over their decision on what to take or what to avoid whilst breastfeeding, expressing their fear of it affecting their infants’ health.

“*I really didn*’*t want to take anything harsh that could affect my baby*’*s health*, *so I was more cautious over what I take. I would say that because I was breastfeeding*, *I was more cautious over what I take and what I eat*, *and I think that using natural herbs would be safer than using chemicals*”. (*BW 18*).

Some participants associated the use of conventional or “Western” medicines as dangerous or harmful to take during breastfeeding. Limited awareness of potential side effects and medicine knowledge had for some women led to the refusal of conventional recommendations for treatment of insufficient breast milk supply, for instance with the prescription medicine domperidone (Motilium^®^).

“*To me*, *it* [*herbal galactagogue*] *seems a lot safer*, *because when I was on Motilium*^®^, *the way how that works changes dopamine levels in the brain which then increases milk supply and prolactin. That to me has always felt like* ‘*Frankenstein*’ *sort of thing. It* [*fenugreek*] *just seems more natural. I am not concerned about any transfer to my milk*, *because it*’*s natural and it has been used for hundreds or thousands of years*, *really*, *I think over time it would have been tested and proven*”. (*BW 7*).

Participants described their concerns over the use of conventional medicine whilst breastfeeding. The general perception of ‘herbal is natural, and natural is safe’ was identified. Many participants displayed a tendency to use herbal alternatives during breastfeeding with the general assumption that herbal galactagogues were safer alternatives compared to other options.

“*I think if you can avoid taking chemical pharmaceutical drugs*, *then I would exhaust every single herbal option before I go near any pharmaceutical*… *because the herbal options are usually much safer*, *they usually don*’*t cause any issues in the baby. For me*, *in any situation I would exhaust any homeopathic or herbal remedy before I went to pharmaceuticals*”. (*BW 11*).

Nevertheless, some women realised that natural remedies may not always be safe and that they would still be mindful and vigilant when choosing their therapy of choice. As one participant commented:

“*Given that it comes from nature*, *even though you can*’*t quantify how much the active ingredient is in there*, *I believe that it may be enough to cause any problems. So in my opinion*, *if a herb or substance is used whilst breastfeeding*, *as long as there is no or significant adverse effect*, *or it can be incorporated to part of your diet*, *then it should be okay*…” (*BW 1*).

The decision and likelihood to use herbal options to promote breastfeeding performance was at times linked to women’s personal preference. Some of the participants appeared to have previously used herbal medicines in managing other health issues separate to pregnancy and lactation. In conjunction with the perception of herbal galactagogues being “safer”, women preferred to use the herbal alternatives during breastfeeding.

“*Even before I was breastfeeding*, *I tend to choose herbal*, *during pregnancy I tend to use herbal and that carried through till I was breastfeeding*, *so that was definitely my first choice*”. (*BW 8*).

#### ***Role and expectations of health care providers***

Breastfeeding women’s expectations of health care providers emerged as a prevailing topic of discussion in these interviews. In the context of this discussion, health care providers included doctors and specialists, midwives, child health nurses, lactation consultants, naturopaths, as well as community pharmacists. Besides health information, expectations of participants centred on drug or product knowledge, including options of alternative therapies, at the same time respecting women’s decision or choice. Participants expected all health care providers to have an adequate level of awareness and knowledge on the availability of all different treatment options. Some of the participants indicated a need for health care providers to be more open-minded, supportive and prepared to provide alternative options should women wish to be able to choose. Participants preferred to receive suggestions or options with information about the available evidence in order to make an informed decision.

“*I think it*’*s the attitude of people. So you know you go and see the lactation consultant*, *and they don*’*t tend to*, *in my experience they don*’*t tend to believe that herbs do very much. Even other health professionals like doctors and pharmacists should be aware or should know about the alternative options*, *so we can make our own decision*, *that would have been very helpful*”. (*BW 5*).

One participant who showed preference to the use of herbal remedies described her experience with health care providers and discontentment after being diagnosed with insufficient milk supply:

“*It* [*using herbal galactagogue*] *is not talked about. Not to boost subject*, *but no one takes these sorts of things seriously I supposed. I think hospitals need to be more open*-*minded and willing to talk about other things other than just manufactured drugs*”. (*BW 2*).

It was also noticeable from the interviews that involving breastfeeding women in decision-making regarding their own health care creates a sense of autonomy which increases the likelihood of adherence to therapy regardless of whether it is conventional or alternative therapies. Many participants believed that information regarding herbal medicines to support breastfeeding should be provided in the information pack supplied at pre-natal clinics.

There was also a perception that many health care providers were not supportive of the use of herbal medicines during breastfeeding, and were not knowledgeable of the range of herbal products available and their evidence in terms of safety and efficacy. Regular users of herbal medicines believed that alternative options should be made available to all breastfeeding women by their health care providers. Some participants further commented on the potential value of awareness in reducing distress and anxiety during early days postpartum.

“*I think people need to know that it does actually work*, *not just some crazy hippy thing. Because actually that was what I thought*, *I initially thought that the herbs were for people who didn*’*t want to use conventional medicines because they have issues with big* “*pharma*” *or whatever*, *but honestly for me*, *it has worked wonders*, *like far better than anything that any doctors have ever recommended it to use. So I just wish that more women actually are aware of it*”. (*BW 7*).

Despite the criticism about the lack of information about herbal remedies from most health care providers, some of the participants did report receiving information and recommendations relating to the use of herbal medicines from these providers. A need for reassurance from health care providers emerged as an underlying theme as some participants elucidated their experiences and relationships with their trusted health care providers. Participants appeared to be comfortable with recommendations from health care providers.

“*I am certainly not opposed to the idea of using herbs during breastfeeding*, *as long as I know and have checked with my child health nurses and doctors or even ringing up a pharmacist*”. (*BW 12*).

“*I never even thought twice about taking it*, *I never had any hesitation in it*, *because of the people who have recommended it to me*, *like the lactation consultants and the naturopaths. I know a naturopath who is very cautious over what she prescribes when you are breastfeeding*, *so I never thought twice. I don*’*t know much of it or how much benefit it had*, *but I have no problems taking it*, *I suppose it is a natural herb*”. (*BW 10*).

The need for research and evidence-based information on the use of herbal medicines during breastfeeding was identified by several participants. They expected health care providers to be up-to-date with the latest research data and be able to translate the information into their daily practice.

“*I guess the supplements out there just need more studies. There*’*s lots of research that goes into glucosamine and fish oil and all these that we think is going to help us*, *but not for breastfeeding*, *it will be nice to have that knowledge to know that it works and it is safe*”. (*BW 3*).

#### ***Parental and peer influence***

The impact of peer and parental influence on breastfeeding women’s decision and choice of therapy was discussed from the perspectives of sources of recommendation and supply. It was evident that some participants were more likely to believe and follow certain recommendations if these were made by parents or peers whom they could relate their experience to or women who had breastfeeding experience.

“ [*The best thing is*] *talking to people on the mothers group page and the breastfeeding support groups*, *they have all walked thought the journey*, *they understand*, *and tried different thing and suggested different options*”. (*BW 4*).

Some participants described feeling stressed from parents and peer pressures to breastfeed as the drive to exploring all available methods to ensure successful breastfeeding. In these instances, potential psychological or emotional benefits of using herbal galactagogues had further benefits in terms of confidence and reassurance.

### Available herbal medicine resources

Three sub-themes were apparent in the interviews as participants described their views on the resources available to them as breastfeeding women regarding the efficacy and safety of herbal medicines.

#### ***Information needs***

Despite their decision to use herbal galactagogues during breastfeeding, the majority of the participants (17 of 20) commented that there was a lack of resources available regarding the use of herbal medicines during breastfeeding. Although these herbal medicines were widely available over-the-counter in Australia, information regarding their efficacy and safety during breastfeeding was perceived as not being well established, or at least not made readily available to them.

“*I don*’*t think there is much out there*, *at least they weren*’*t easily available. If it wasn*’*t for my friend at mothers*’ *group*, *I wouldn*’*t have known to take fenugreek*”. (*BW 12*).

“…*I think we can do with a lot more information and make it more widely available so that people who are in need of it can use them*”. (*BW 8*).

The majority of participants expressed a need for accessible evidence-based information and more research to be conducted to facilitate safe and effective use of medicines during breastfeeding to promote successful breastfeeding and avoid unnecessary early cessation of breastfeeding.

“*If it was proven medically and endorsed*, *more people would be able to use that* [*herbal galactagogue*], *instead of just giving up breastfeeding when they feel the supply is low or grabbing the first bottle of formula*”. (*BW 18*).

In addition to the general need for further research, a need for research specifically conducted locally was also identified to facilitate the application of findings to the Australian health care context. As herbal preparations exist in various brands and dosage formulations throughout the world, some participants found it impractical to relate to information or studies conducted overseas.

“*I think it*’*s better if we have our own*, *because obviously in all countries the culture is different*, *obviously you can*’*t do research in every single country*, *but I think I will be more incline to believe it if it was from Australia rather than from overseas. Also different countries have different brands*, *which to me in my case*, *different brands gave me a different effect. So like for example if you see a brand from an American website*, *but then you may not get it in Australia. I think it is really important to have an Australia sort of research around it*”. (*BW 7*).

#### ***Credibility and reliability of information***

Many participants relied on the internet or their friends and family for information, advice and recommendations. Unknown credibility and reliability of information accessed from the internet was highlighted during the interviews. Although many participants questioned the trustworthiness of information obtained from non-accredited sources, they were left with no other option but to use the internet.

“*Most of the stuff you get from the internet*, *I am just always worried about reliability*, *and the more you read*, *you tend to trust it more*, *which may not necessarily be a good thing sometimes*”. (*BW 5*).

Participants further cited the need for reliable information to be endorsed by organisations such as the Australian Breastfeeding Association (ABA).

“*I had to rely on* [*internet*] *forum discussions and word of mouth to make up my mind whether a herbal product is suitable for me or not. So if it was endorsed by a medical* [*group*] *or I know the Australian Breastfeeding Association is not a medical*, *but if it was endorsed more medically in some way or another*, *it might be more helpful*, *people might be able to use it more*”. (*BW 17*).

Along with the general perception of the lack of easily accessible reliable information, one participant who was both a health professional and a mother of an eight-month old at the time of the interview, described her views on the information resources. Inconclusive information was seen as confusing and further posed a dilemma:

“*If there is something available*, *I find that it is usually inconclusive. There is just not enough data and they* [*the resources*] *leave the ball to the mothers*’ *court to decide whether they want to take it. I feel like there is no conclusive information as such regarding herbal medicines during breastfeeding*”. (*BW 1*).

From the perspectives of some participants, reliance on parents or close family members and friends for breastfeeding-related information was considered sufficient.

“*I only went to where my mum told me*, *so I personally never looked into safety and efficacy of herbal medicines during breastfeeding*”. (*BW 1*).

#### ***Expectations of health professionals***

In general, health professionals were viewed as reliable sources of information. Besides voicing their need for additional research studies, participants also demonstrated a desire for written and verbal information from their health care professionals with regards to the use, safety and efficacy of herbal medicines during breastfeeding. It appeared that some participants perceived that there was a lack of health professionals’ awareness about the availability of evidence-based information regarding use of herbal medicines during breastfeeding. Participants expected information related to the use of herbal medicines to be provided in a leaflet or pamphlet format by their health professionals.

“*The info packs that you get from the hospitals and pamphlets from nurses don*’*t have much information about herbal remedies for use during breastfeeding. Like I mentioned earlier*, *if I had known the presence of fenugreek for example*, *I would have tried that with my first child and may have a better or easier time with breastfeeding*”. (*BW 6*).

Acknowledging that there is a lack of available information, participants believed that health professionals should endeavour to provide guidance. Information on herbal options during breastfeeding should be made readily available to all breastfeeding women as suggested by some participants.

“*At least on what*’*s available*, *I mean there might not be a lot of information available*, *but at least to guide us on where to look out for information*”. (*BW 5*).

Despite expecting health professionals to have adequate levels of knowledge, participants had varying expectations of different health professionals:

“*I find people who are working in natural health are more comfortable than people who are working in main stream health*, *they are more hesitant to recommend stuff*, *they have a more complex*… *I mean they couldn*’*t really say whether something was safe or not*”. (*BW 8*).

In the context of reliable information resources, community pharmacy was perceived as an easily accessible health destination and pharmacists were recognised by participants as overall medicine experts.

“…*the few times when I had questions about medications during breastfeeding*, *I called up the pharmacies and they were fantastic*”. (*BW 9*).

### Level of breastfeeding support

A multidisciplinary team consisting of a diverse group of health care providers including doctors, pharmacists, child health nurses, midwives and lactation consultants was seen by participants as contributors to the current health care system. Many of the health care providers were mentioned throughout the interviews when participants described their experiences and understandings with “health care” and “breastfeeding support”. Despite her perception of insufficient information available regarding the use of herbal medicines during breastfeeding, one participant acknowledged the level of assistance and support provided:

“*I would say there is certainly a lot of help out there*, *I mean a lot of breastfeeding help. You have the child health nurses*, *midwives*, *lactation consultants*, *chemists*, *doctors and all to help and ask questions if you need to*, *but I don*’*t think there is enough information out there about use of herbal medicines*”. (*BW 15*).

Despite some positive experiences, participants highlighted several areas for improvement. Immediate postpartum and early parenthood were viewed as challenging and may be associated with anxiety, stress and confusion in some women, which may impact on their ability to take in any information. According to some participants, in-depth practical information regarding breastfeeding were not provided until immediate postpartum. Although some pre-natal classes may have touched on the subject, the level of breastfeeding-related information was viewed as insufficient to enable a full understanding and anticipation of the potential breastfeeding-related issues which women may encounter. Hence, participants suggested that breastfeeding and related information be part of the focus during pre-natal classes or information sessions to avoid confusion during the lactation stage.

“*Before birth*, *because that*’*s when you want to learn as much as possible about what*’*s going to happen when the baby comes because you have no idea. But after baby arrives*, *you are so consumed and you don*’*t pay attention to what*’*s happening around you. Especially*, *I didn*’*t really remember anything during the first week. So doctors and nurses could have told me things but I wouldn*’*t remember*, *but information should be available before birth*”. (*BW 2*).

Many of the participants were not aware of insufficient breast milk supply being a potential issue before the baby was born. Besides the provision of breastfeeding-related information during pre-natal classes, participants further suggested that potential issues with insufficient breast milk supply and information on options available to boost supply, including herbal galactagogues and non-pharmacological therapies should be made available prior to delivery. Pre-natal classes and visits to health care providers were proposed as the most appropriate avenues. This valuable information was perceived as potentially useful and expedient when discussed before the birth of their infants, especially for first-time new parents.

“*I only found out that* [*fenugreek*] *after it* [*low milk supply*] *became an issue. We are told the importance of breastfeeding and all that*, *but not really about being aware that supply could be an issue. So*, *if this was discussed earlier on before delivery*, *we can prepare and start taking some supplements*, *rather than addressing it way down the track when you realized that you have a supply issue*”. (*BW 9*).

“*Most mothers will be going to prenatal classes before the baby is born. They do explain breastfeeding but they don*’*t go through the different things that you can do to help the situation. They sort of tell you this is how you should breastfeed to get the attachment right and things like that*, *but not really like the issues that could come with it. They can prepare themselves if it didn*’*t happen the way it was meant to*”. (*BW 15*).

“*Sometimes it is so*, *you are so sleep deprived*, *when the baby arrived*, *you are thinking*, *I am just trying to keep my head above water*, *so it*’*s great to have information out there before the baby arrives*, *or particularly with your first baby. I think maybe they should have given you a little bit more information on breastfeeding and other options out there if you have problems with supply*, *as well*”. (*BW 16*).

To avoid confusion, it is also crucial that all health care providers across the multidisciplinary team are up-to-date with recommended guidelines to ensure consistent and dependable information be delivered.

“*I don*’*t think that there*’*s*, *this is my opinion*, *there isn*’*t a massive amount of support or in hospital for my first baby. I was told different things in regards to breastfeeding*, *different ways*, *it was just confusing*, *so I had to work out how it would work for me*, *and that*’*s very common in first time mum. They are told different things and ended up feeling very confused*”. (*BW 16*).

## Discussion

Although many previous studies have examined the perspectives of women towards the use of medications during breastfeeding, most had focused on the use of conventional medications [22]. To address the research gap, this study focused on the perspectives and attitudes of this population towards the use of herbal galactagogues.

All of the participants of this study appreciated and valued the benefits of breastfeeding and supported the ‘breast is best’ philosophy. Participants showed a positive attitude towards breastfeeding and were willing to make efforts necessary to ensure the success of breastfeeding, including the use of herbal medicines to promote breastfeeding performance. For most participants, the strong desire to continue to breastfeed accompanied by recommendations from family and friends had led to the use of herbal galactagogues during breastfeeding.

The potential psychological or emotional impact of using herbal galactagogues during breastfeeding should not be underestimated. As evident in this study, confidence and self-empowerment emerged as an over-arching theme throughout the interviews, especially when participants described their positive experiences. This finding was also observed in a similar study conducted by Westfall, who highlighted the positive feedback provided by her participants despite the lack of scientific evidence for most herbal galactagogues’ efficacy [14].

It is not surprising that women who had used herbal galactagogues during breastfeeding had accepted and adopted an integrative and holistic approach for their own healthcare. Many studies have demonstrated the acceptance and practice of integrative healthcare amongst the general Australian population [23-26]. Some women who had used herbal galactagogues during breastfeeding used herbal medicines prior to pregnancy and lactation. Consistent with previous research on perspectives of alternative health care users, this finding demonstrated that some women chose alternative therapies to gain autonomy and a sense of control over their own health, and that the decisions are accustomed to their values and beliefs [27-31]. The sense of empowerment when a breastfeeding woman actively seeks out and adheres to an alternative regimen plays a positive role in her breastfeeding journey. As perceived insufficient milk supply is one of the major causes of premature cessation of breastfeeding, the use of herbal galactagogues provides a sense of empowerment and self-efficacy which may aid in overcoming the issue [32].

Some of the participants were concerned about adverse effects and the risk of harming their infants with the use of conventional medicines. Despite this concern, considering the popularity and acceptance of integrative or holistic healthcare in the general population, some women may be likely to opt for alternative options, for example herbal remedies, to promote breastfeeding performance. Previous dissatisfied experience with conventional treatments may also encourage the use of alternative therapies whilst breastfeeding [27]. Due to the potential of misperception by some women that herbal remedies are always safe, the public should be encouraged to consult health professionals prior to using any medicine whilst breastfeeding.

Consistent with the literature and previous findings, breastfeeding women identified the need for more in-depth information, including scientific evaluation of the efficacy and safety of herbal galactagogues and other herbal medicines during breastfeeding [22,28,33]. Women expect health professionals to have adequate knowledge and to be willing to offer advice and discussion over alternative therapies to promote breastfeeding performance. By acquiring up-to-date knowledge and involving women in decision-making, health professionals may help to promote compliance and success of breastfeeding-related therapies. Further research into the safety and efficacy of herbal galactagogues and ongoing education about CAMs will enable health professionals to be equipped with the knowledge to meet the expectations of the public.

Although none of the questions in the interview guide was designed to address this topic, breastfeeding women’s perspectives on the current health care system and the lack of breastfeeding support provided emerged as a common focus of discussion by some participants as they described their experiences before, during and after delivery. A mix of positive and negative feedback were noted from the narrative conservation during the interviews.

### Limitations

As the nature and process of recruitment involved recruiting participants from naturopathic clinics initially, and all participants self-volunteering to be interviewed, there is a possibility for some degree of bias in the sample selection. Recruiting participants from naturopathic clinics increases the likelihood of recruiting women with similar positive perspectives and attitudes towards the use of herbal galactagogues. Participants of this study were self-selected and hence are not likely to be a true representative sample of all breastfeeding women in Australia, nor a representation of all breastfeeding users of herbal galactagogues. As with all qualitative studies, a known limitation is the challenge in attempting to generalise findings to the wider population, as at times, the findings may be unique only to the specific participants [34]. Nevertheless, this study has enhanced our understanding of women’s perspectives and factors which may influence their choice of therapy whilst breastfeeding.

## Conclusions

This qualitative study provides insight into the perspectives and attitudes of breastfeeding women towards the use of herbal galactagogues. The positive attitudes of herbal galactagogue users should prompt health professionals and researchers to further explore this topic whilst the negative views regarding timing of education on breastfeeding and inconsistency of information should be taken into consideration to improve services for breastfeeding women. Further research into the safety and efficacy of herbal galactagogues, including clinical trials and case reports, are urgently required to provide research-based evidence to inform health professionals and breastfeeding women.

## Competing interests

The authors declare that they have no competing interests.

## Authors’ contributions

TFS conducted and analysed the data as part of her PhD degree. TFS, JS and LT were responsible for the design of the study and developed the interview guide. TFS and LH were responsible for data analysis. TFS, LH, JS and LT all contributed to writing of the manuscript. All authors read and approved the final manuscript.

## Pre-publication history

The pre-publication history for this paper can be accessed here:

http://www.biomedcentral.com/1472-6882/14/216/prepub
